# Intestinal expression of metal transporters in Wilson’s disease

**DOI:** 10.1007/s10534-013-9668-5

**Published:** 2013-08-21

**Authors:** Adam Przybyłkowski, Grażyna Gromadzka, Adriana Wawer, Tomasz Grygorowicz, Anna Cybulska, Anna Członkowska

**Affiliations:** 1 Department of Clinical and Experimental Pharmacology, Medical University of Warsaw, ul. Krakowskie Przedmieście 26/28, 00-927 Warsaw, Poland; 2 Department of Gastroenterology, St Ann’s Hospital, Warsaw, Poland; 3 2nd Department of Neurology, Institute of Psychiatry and Neurology, Warsaw, Poland

**Keywords:** Wilson’s disease, Metal transporters, Intestine, Copper, ATP7A, DMT1, CTR1, ATP7B

## Abstract

In Wilson’s disease (WND), biallelic *ATP7B* gene mutation is responsible for pathological copper accumulation in the liver, brain and other organs. It has been proposed that copper transporter 1 (CTR1) and the divalent metal transporter 1 (DMT1) translocate copper across the human intestinal epithelium, while Cu-ATPases: ATP7A and ATP7B serve as copper efflux pumps. In this study, we investigated the expression of CTR1, DMT1 and ATP7A in the intestines of both WND patients and healthy controls to examine whether any adaptive mechanisms to systemic copper overload function in the enterocytes. Duodenal biopsy samples were taken from 108 patients with Wilson’s disease and from 90 controls. CTR1, DMT1, ATP7A and ATP7B expression was assessed by polymerase chain reaction and Western blot. Duodenal CTR1 mRNA and protein expression was decreased in WND patients in comparison to control subjects, while ATP7A mRNA and protein production was increased. The variable expression of copper transporters may serve as a defense mechanism against systemic copper overload resulting from functional impairment of ATP7B.

## Introduction

Wilson’s disease (Online Mendelian Inheritance in Man [OMIM] 277900, WND) is an inherited autosomal recessive metabolic disorder characterized by impaired copper metabolism. The affected gene is *ATP7B,* which encodes an intracellular membrane-spanning P-type adenosine triphosphatase (ATPase), a copper transporting protein abundant in hepatocytes (Frydman et al. 1985). The presence of pathogenic mutation in both alleles of *ATP7B* gene results in impaired transport of copper from hepatocytes into the bile. Since excretion with bile is the main route of copper removal from the body, dysfunction of ATP7B triggers accumulation of copper within the liver, brain and other parenchymal structures. Copper overload leads to organ damage and the clinical manifestations of WND.

A few compensatory mechanisms have been described in WND patients. One of them is increased renal excretion of copper (Cartwright et al. 1954). The other proposed backup mechanisms are: loss of enterocytes with sequestered copper and copper loss with increased production of digestive secretions (Linder 2002). The wall of the gastrointestinal tract is the gateway for copper; thus, adaptive changes which reduce copper absorption from the gastrointestinal tract may be expected in the intestinal epithelia of WND patients. In polarized epithelia of the intestine, copper transport is accomplished by two separate steps: inflow from the lumen into the enterocyte and efflux from the cell into the blood. There are two major candidates for mediating apical copper uptake: copper transporter 1 (CTR1) and divalent metal transporter 1 (DMT1) (Lee et al. 2002, Arredondo et al. 2003; Zimnicka et al. 2007). Alternative copper absorption pathways have been also proposed, including anion exchangers, endocytosis, and sodium-dependent amino-acid transporters (Zimnicka et al. 2011). The mechanism of copper efflux from the enterocyte has been better defined. It includes two proteins: ATP7A and ATP7B, both belonging to the P-type ATPase family of proteins translocating copper across cellular membranes in an adenosine triphosphate (ATP)-dependant manner (Gupta and Lutsenko 2009). ATP7A mediates copper transfer across the basolateral membrane of the enterocytes towards the blood. The ATP7B protein has been proposed as a modifier of intestinal copper absorption via copper excretion through the apical surface of the enterocyte and/or via vesicular sequestration (trapping) of copper within the cell (Gupta and Lutsenko 2009).

The adaptive changes of the intestinal epithelium in WND subjects may include differential expression of copper importers, as well as variable expression of proteins involved in copper efflux from the enterocyte. The aim of this study was to evaluate the expression of metal transporters, i.e. DMT1, CTR1, ATP7A and ATP7B, in the duodenal mucosa of WND patients and controls. We also investigated both if the expression of copper transporters in the intestine is related to copper metabolism parameters and whether it is modulated by anti-copper treatment.

## Patients and methods

### Patients

One hundred and eight patients with WND were recruited onto the study (38 treatment-naive patients and 70 patients on de-coppering therapy (zinc salts or D-penicillamine)). The control group comprised patients diagnosed as a result of dyspeptic symptoms in outpatient clinics. WND diagnosis was made according to the scoring system for the diagnosis of WND based on clinical signs and symptoms, evidence of impaired Cu metabolism (abnormal test results for: serum ceruloplasmin concentration, serum Cu concentration, and Cu excretion in urine), the presence of the Kayser–Fleischer ring, as well as the presence of pathogenic mutations in both alleles of the *ATP7B* gene (Ferenci et al. 2003).

In all study participants, oesophagogastroduodenoscopy with a duodenal biopsy was performed. Peripheral blood samples were collected before endoscopy. The samples once collected were immediately sent to the laboratory for testing or were frozen at −80 °C for further analysis.

The study protocol conforms to the ethical guidelines of the 1975 Helsinki Declaration and was approved by the ethics committee at the Institute of Psychiatry and Neurology, Warsaw, Poland. All patients gave informed written consent to participate in the study.

### Biochemical investigations of copper metabolism

Serum samples were analyzed for copper concentration by flame atomic absorption spectroscopy, and serum ceruloplasmin was measured by the Ravin method (1961).

### Real-time quantitative polymerase chain reaction (PCR)

Total RNA was isolated form duodenal samples using Tri-Reagent BD (Sigma-Aldrich, Poznan, Poland) according to the manufacturer’s protocol. RNA pellets were re-suspended in 12 μl ribonuclease free water (Eppendorf, Hamburg, Germany) and incubated for 10 min at room temperature to solubilize RNA. RNA content and purity were determined spectrophotometrically. Reverse transcription was carried out with 1.5 μg RNA using a High Capacity cDNA Reverse Transcription Kit with RNAse Inhibitor (Applied Biosystems, Carlsbad, USA) according to the manufacturer’s instructions. Real-time PCR was used to determine gene expression levels using TaqMan (Applied Biosystems, Carlsbad, USA) technology on an OPTICON2 sequence detection system (MJ Research, Waltham, USA). Primers were designed to span exon–exon junctions to exclude the detection of genomic DNA. The following transcripts were assayed: SLC31A1 (Hs00741015_m1), SLC11A2 (Hs00167206_m1) ATP7B (Hs00163739_m1), and ATP7A (Hs00163707_m1) (Applied Biosystems, Carlsbad, USA). Fluorescence emission during the PCR reaction was continuously monitored to determine the threshold cycle (C_T_). The housekeeping gene hypoxanthine phosphoribosyltransferase 1 (*HPRT*) was chosen as the reference standard to normalize patient samples for RNA quality and quantity (the TaqMan Gene Expression Assay Hs02800695_m1 (Applied Biosystems, Carlsbad, USA) was used)). Experimental samples were assayed simultaneously with positive and negative (no reverse-transcription template) controls on the same plate. All samples were tested in duplicate and the results were averaged. The relative expression of the target genes in experimental samples was calculated using the efficiency-calibrated Pfaffl method and has been presented in arbitrary units (AU) calculated by dividing the mRNA expression of the studied gene/the reference gene (Pfaffl et al. 1998).

### Western blot analysis

Intestine samples were homogenized in 120 μl of radio-immunoprecipitation assay (RIPA) lysis buffer containing 50 mM Tris pH 7.4, 150 mM NaCl, 2 mM ethylenediaminetetraacetic acid, 1 % NP-40, 0.25 % Na-deoxycholate, 1 mM phenylmethanesulfonylfluoride, and 1 mM Na_2_VO_3_ in the presence of a protease inhibitor cocktail (P8340, Sigma-Aldrich, Poznan, Poland). Samples were incubated on ice for 40 min, then centrifuged at 10,000 × *g* for 15 min. Supernatants were collected, and protein concentrations were determined by the Bradford method (1976). Membrane proteins (65 μg) were electrophoresed through polyacrylamide gels (CTR1 and DMT1, 10 %; ATP7A and ATP7B, 8.5 %) and transferred at 30 mA at 4 °C overnight onto a nitrocellulose membrane. Equal amounts of recombinant ATP7A, ATP7B, or DMT1 (Abnova, Taipei, Taiwan) were loaded onto the gels as standards and controls for antibody specificity; HeLa whole-cell lysate (Santa Cruz Biotechnology, Dallas, USA) was used for this purpose with CTR1. Membranes were stained with Ponceau S solution (Sigma-Aldrich, Poznan, Poland) to verify equal loading and protein transfer. Non-specific binding to the membrane was blocked for 1 h at room temperature with 5 % nonfat milk in Tris-buffered saline with Tween 20 (TBST) buffer. Blots were incubated overnight with CTR1 (Santa Cruz Biotechnology, Dallas, USA), DMT1, ATP7A, or ATP7B (Abnova, Taipei, Taiwan) primary antibody (1:1400–2000 dilution in 5 % nonfat milk in TBST) and then washed three times with TBST. Donkey anti-rabbit Ig horseradish peroxidase-linked antibody (Amersham/GE Healthcare, Freiburg, Germany) was used as the secondary antibody (1:10.000 in 5 % nonfat milk in TBST). Processed blots were exposed to X-ray film for the optimum exposure time. Bands were detected with the ECL Plus system (Amersham/GE Healthcare, Freiburg, Germany), and quantified with ImageJ 1.46 (http://rsbweb.nih.gov/ij/) after normalization of the signal to β-actin or glyceraldehyde-3-phosphate dehydrogenase (GAPDH). The relative expression of the examined sample was presented in AU calculated by dividing the optical density of the sample by the optical density of the standard protein.

### Immunohistochemistry

The duodenal specimens were fixed in neutralized 4 % paraformaldehyde immersed in 70 % (v/v) ethanol at 4 °C overnight, embedded in paraffin, and sectioned at a thickness of 4 μm. In preparation for staining, slides were de-paraffinized with xylene and hydrated through immersions in graded ethanol. Heat-induced antigen retrieval was performed by heating in citrate buffer, at pH 6. Thereafter, samples were subjected to 3 % (v/v) H_2_O_2_ in PBS for 15 min, blocked with 3 % (w/v) bovine serum albumin in PBS for 30 min, and incubated with primary antibodies (LSBio, Seattle USA) overnight at 4 °C. Secondary antibody incubation and detection were performed with EnVision + System HRP (Dako, Glostrup, Denmark). After immunohistochemical staining, tissues were counterstained with Mayer’s hematoxylin.

### Statistical analysis

Data were analyzed using STATISTICA 9.0 (StatSoft, Cracow, Poland). Normally distributed variables (AU of mRNA expression and protein concentration) are presented as means and standard deviations (SD). Variables that were not normally distributed in the analyzed population (age, serum copper and serum ceruloplasmin concentrations) are presented as medians and interquartile ranges (IQR). The student’s *t* test with unequal variances and the Mann–Whitney U test were used for analysis of normally and non-normally distributed variables, respectively. Categorical variables were compared between groups with the χ^2^ test (with Yates correction, if appropriate). Relationships between variables were examined using regression analysis. The significance level was set at 0.05.

## Results

### General patient characteristics

The clinical characteristics of the groups participating in the study are presented in Table 1. Patients with WND are characterized by significantly lower serum concentrations of copper (33.5 [IQR 51.0] μg/dl vs. 111.0 [33.0] μg/dl; *p* < 0.000000) and ceruloplasmin (5.7 [IQR 11.6] mg/dl vs. 34.3 [10.8] mg/dl; *p* < 0.000000) compared to the control group (Table 1). WND patients on de-coppering therapy had lower serum copper concentrations (23.5 [IQR 46.0] μg/dl vs. 45.5 [37.0] μg/dl; *p* < 0.003) and lower ceruloplasmin concentrations (5.4 [IQR 10.9] mg/dl vs. 9.2 [13.6] mg/dl; *p* < 0.04) than those who were treatment-naive.

**Table 1:** Clinical characteristics of study participants

	WND patients (*n* = 108)				Controls (*n* = 90)	*p* value
	treatment naive *N* = 38		treated *N* = 70	all		
Age, years; median (IQR)	28.5 (16.0)		30.5 (15.0)	30.0 (15.0)	41.5 (29.5)	a < 0.0001 b < 0.0001 c < 0.000004 d < 0.3
Women/men; n (%)	19/19 (50/50)		36/34 (51/49)	55/53 (51/49)	60/30 (67/33)	a < 0.07 b < 0.5 c < 0.02 d < 0.9
Serum copper, μg/dL; median [IQR]	45.5 [37.0]		23.5 [46.0]	33.5 [51.0]	111.0 [33.0]	a < 0.000000 b < 0.000000 c < 0.000000 d < 0.003
Serum ceruloplasmin, mg/dl; median [IQR]	9.2[13.6]		5.4[10.9]	5.7 [11.4]	34.3[10.8]	a < 0.000000 b < 0.000000 c < 0.000000 d < 0.04

Reference range values: ceruloplasmin: 25–45 mg/dl; copper: 70–140 μg/dl. *p* values were determined with χ^2^ test or the Mann–Whitney *U* test as appropriate, *p* value in the last column is given for the following comparisons: a—treatment naive WND and controls, b—WND patients on treatment and controls, c—all WND patients and controls, d—WND patients treatment naive and WND patients on treatment

*WND* Wilson’s disease, *IQR* interquartile range

### The duodenal expression pattern of copper transporting proteins in WND patients and in controls

**Fig. 1:**
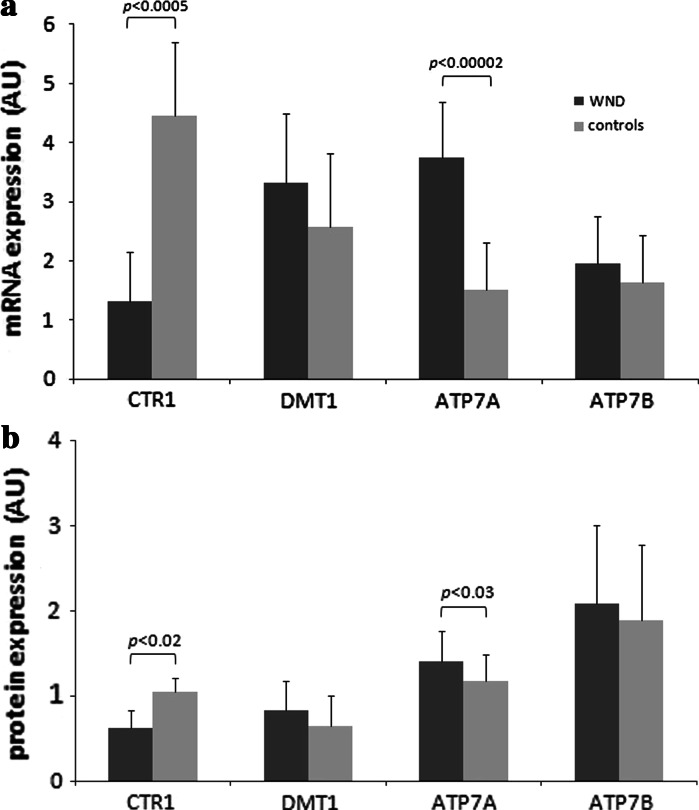
Messenger RNA expression for *CTR1*, *DMT1*, *ATP7A*, and *ATP7B* genes **a** and CTR1, DMT1, ATP7A, and ATP7B protein expression **b** in duodenal samples from Wilson’s disease (WND, *n* = 108) patients and controls (*n* = 90). The relative expression of the target genes was calculated using the efficiency-calibrated Pfaffl method and has been presented in arbitrary units (AU), relative expression of proteins represents the ratio of optical density of the examined protein versus standard protein. Data are presented in arbitrary units (AU) as means and standard deviations. *p*-values for comparisons between WND patients and controls were calculated with the two tailed t-test

**Fig. 2:**
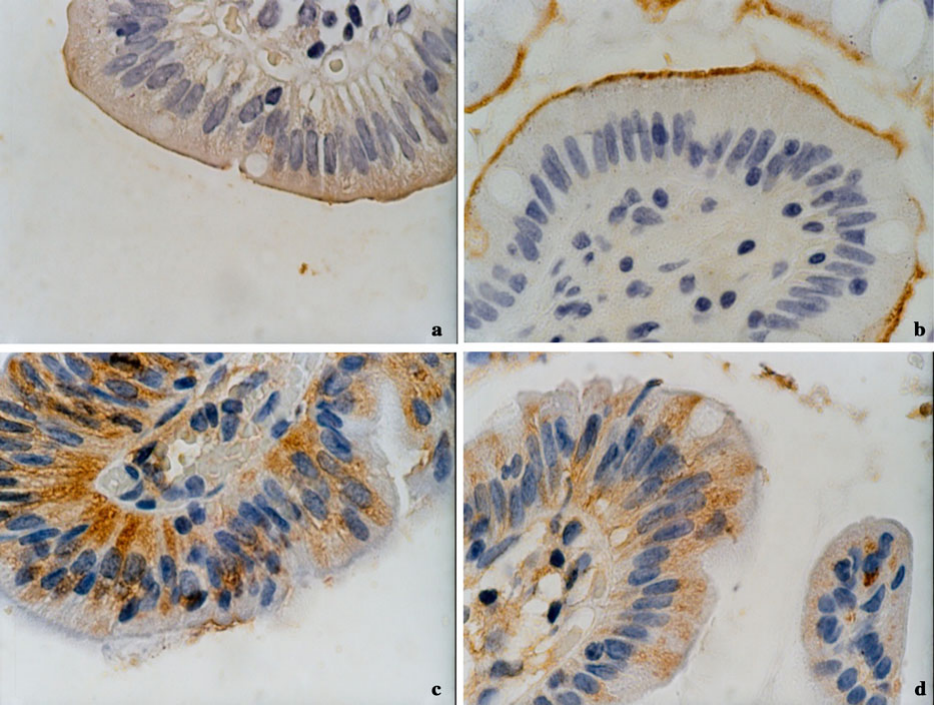
Immunohistochemical analysis of duodenal samples of Wilson’s disease patients. Tissues were probed with antibodies against CTR1 **a**, DMT1 **b**, ATP7A **c**, or ATP7B **d**. Magnification × 1000. Localization of the proteins and staining intensity was similar in Wilson’s disease patients and controls (data not shown)

The duodenal expression of *CTR1* mRNA was approximately three-fold lower in WND patients compared with controls (AU: 4.5 [SD 0.8] vs. 1.3 [1.2]; *p* < 0.0009; Fig. 1a). The duodenal CTR1 protein concentration was also lower in WND patients than in healthy subjects (AU 0.6 [SD 0.3] vs. 1.1 [0.2]; *p* < 0.02) (Fig. 1b). In the immunostained sections of duodenal samples, in both groups, the CTR1 protein was visible throughout the cytoplasm, with the highest density at the luminal surface of enterocytes (Fig. 2a).

The levels of *DMT1* mRNA and protein expression did not differ between WND patients and controls (Fig. 1 a–b). In both groups, immunohistochemical analysis detected DMT1 proteins localized at the brush border of the enterocytes (Fig. 2b).

WND patients were characterized by significantly higher duodenal expression of *ATP7A* mRNA (AU: 3.8 [SD 0.9] vs. 1.5 [0.8]; *p* < 0.00002) and ATP7A protein (RE: 1.4 [SD 0.3] vs. 1.2 [0.3]; *p* < 0.02) than healthy controls (Fig 1 a–b). In both groups, immunohistochemistry revealed basolateral ATP7A distribution within the enterocyte (Fig. 2c).

No significant difference was detected in duodenal *ATP7B* mRNA expression between WND patients and controls (Fig. 1 a–b); in both groups, ATP7B protein localized throughout the cytoplasm of the enterocytes (Fig. 2d).

### The anti-copper treatment and the duodenal expression of metal transporters

**Fig. 3:**
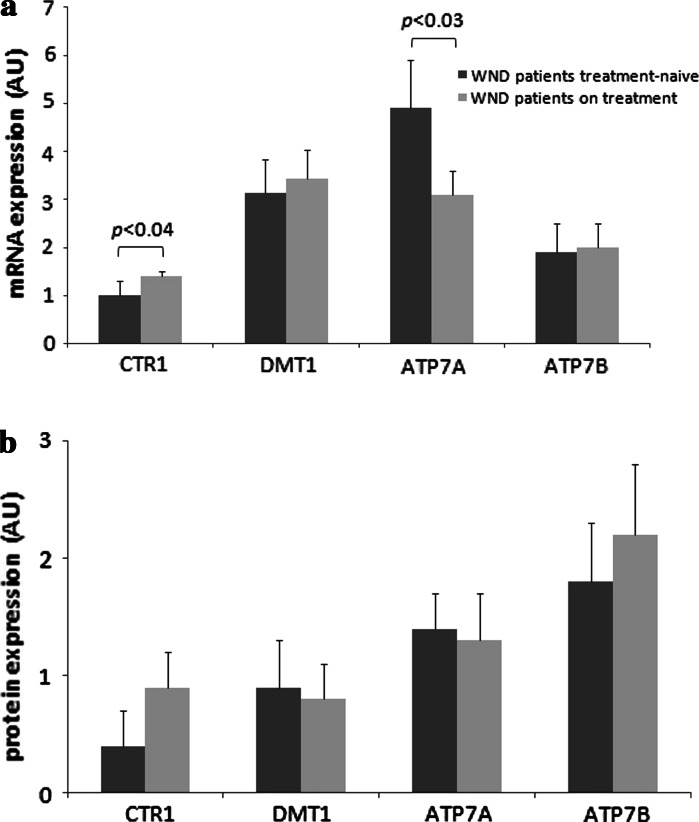
Messenger RNA expression for *CTR1*, *DMT1*, *ATP7A*, and *ATP7B* genes **a** and CTR1, DMT1, ATP7A, and ATP7B protein expression **b** in duodenal samples taken from treatment naive Wilson’s disease (WND) patients (*n* = 38) and WND patients on treatment (*n* = 70). The relative expression of the target genes was calculated using the efficiency-calibrated Pfaffl method and has been presented in arbitrary units (AU), relative expression of proteins represents the ratio of optical density of the examined protein versus standard protein. Data are presented in arbitrary units (AU) as means and standard deviations. *p*-values for comparisons between WND patients and controls were calculated with the two tailed t-test

The CTR1 mRNA expression was slightly lower in treatment-naive patients than in patients on de-coppering therapy (AU: 1.0 [SD 0.3] vs. 1.4 [0.1]; *p* < 0.04) (Fig. 3a). No significant difference in the corresponding protein expression was detected (Fig. 3b).

Treatment naive WND patients were also characterized by higher *ATP7A* mRNA levels compared with WND patients undergoing active anti-copper treatment (AU: 4.9 [SD1.0] vs 3.1 [0.3]; *p* < 0.03, Fig. 3a). ATP7A protein expression did not differ significantly between groups (Fig. 3b).

No significant difference in the mRNA and protein expression of DMT1 and ATP7B was noticed between WND patients treated or untreated with anti-copper drugs (Fig. 3 a–b).

### The duodenal expression pattern of metal transporters and biochemical copper metabolism parameters

In WND patients and controls, the duodenal mRNA and protein expression of all the proteins studied (CTR1, DMT1, ATP7A, ATP7B) did not correlate with the serum concentration of copper metabolism parameters (data not shown).

## Discussion

To the best of our knowledge, the current study is the first ever that has aimed to evaluate the expression of proteins involved in copper absorption as well as in copper efflux from the enterocytes in WND patients. Messenger RNA and protein expression were studied for four proteins–CTR1, DMT1, ATP7A and ATP7B–in duodenal samples taken both from WND patients and from controls.

The first important observation from this study seems to be the down-regulation of CTR1 in the enterocytes of WND patients as compared to healthy controls. CTR1 plays a crucial role in dietary copper absorption in mammals (Nose et al. 2010); however, there are some controversies regarding CTR1 localization and function in the intestinal epithelium (Gupta and Lutsenko 2009). It has been proposed that the CTR1 protein serves as an apical copper transporter responsible for copper absorption from the lumen of the gastrointestinal tract (Nose et al. 2010), an intracellular copper transporter (Kuo et al. 2006), and/or the basolateral membrane transporter carrying bioavailable copper from the bloodstream into the enterocyte to satisfy its own needs (Zimnicka et al. 2007, Gupta and Lutsenko 2009). In the specimens taken from the patient and controls populations we examined, CTR1 expression occurred mainly near the apical membrane of the enterocytes. The same localization, reflecting the dietary copper absorption function of CTR1, has been observed in other mammalian species (Nose et al. 2010).

The decreased transcription activity of *CTR1* and the low CTR1 protein production in WND patients that we have documented may be a plausible adaptative mechanism, which aims to reduce copper influx into enterocytes. Our results seem to be in line with previous observations in mice with selective *Atp7b* gene knockout in the liver (an animal model of WND) (Gray et al. 2012). These mice presented low *Ctr1* expression in hepatocytes as compared to wild littermates. The down-regulation of Ctr1 in *Atp7b* ^−/−^ mice results in decreased copper retention within the liver.

In addition to diminished expression of CTR1, we have also observed increased ATP7A expression in WND patients. The main function of ATP7A in enterocytes is the transport of copper to cuproenzymes, the sequestration of copper into vesicles and copper efflux from enterocytes to the basolateral space (Ravia et al. 2005, Rossi 2005, La Fontaine and Mercer 2007, Nyasae et al. 2007). The crucial role of ATP7A in intestinal copper homeostasis is documented by the phenotype of Menkes disease (OMIM 309400), an inherited X-linked dysfunction of ATP7A (Menkes et al. 1962). Affected individuals display copper accumulation in enterocytes, with a subsequent deficiency of this metal in other cells (Kaler 1994). The intestinal expression of ATP7A proteins increases in response to copper overload (Ravia et al. 2005). Bauerly et al. 2005showed that, in the intestines of suckling rat pups fed with copper-rich milk, ATP7A mRNA and protein levels were significantly higher in comparison with animals on a standard diet. Augmented ATP7A expression was accompanied by increased copper retention in the intestine of the young rats. Based on the aforementioned results, it seems likely that upregulated ATP7A production in the intestine of WND patients serves to trap copper in the enterocyte and/or to stimulate its efflux outside the cells.

The increased ATP7A and diminished CTR1 expression in enterocytes of WND patients suggests the presence of a copper-sensitive mechanism regulating the expression of copper transporters. Based on the results of published studies, it seems that there are at least two such mechanisms: local and systemic. The local one has been characterized in human cancer cells. In those cells, production of CTR1 is controlled by the binding of a transcription factor Sp1 to the GC boxes located in the vicinity of the *CTR1* promoter (Song et al. 2008, Liang et al. 2012). Sp1 itself is regulated by copper concentration, too. In cases of copper sufficiency, copper is bound to Sp1, which prevents its binding to the *CTR1* and *Sp1* promoters. Conversely, low copper levels upregulate Sp1 and CTR1 expression. Several Sp-like binding sites are present in the promoter of a murine ATP7A orthologue (Liwei Xie 2012). The aggregate of these data suggests that Sp1 is a candidate regulator of copper homeostasis in humans. The variable expression of ATP7A and CTR1 in the intestine of WND patients could also result from a systemic signal from copper overloaded organs. For iron, which is closely related to copper and where the metabolic pathways are interspersed with copper pathways, there is a clear regulatory mechanism based on the hepatic hormone hepcidin. Hepcidin, in response to a high iron load, inhibits iron transport in enterocytes by decreasing iron transporter expression (Rossi 2005). In mice with cardiac specific knockout of *Ctr1* (Ctr1^hrt/hrt^) increased serum Cu levels, a decrease in hepatic Cu load and increased expression of ATP7A in the intestine and liver have been documented (Kim et al. 2010). Serum taken form Ctr1^hrt/hrt^ mice was able to stimulate ATPTA expression in the culture of human primary umbilical vein endothelial cells. These observations suggest the existence of the systemic regulation of copper homeostasis. The copper content in the duodenal samples we collected from patients and controls was below the detection threshold of atomic absorption spectrophotometry; similar to the results of the other authors (Sturniolo et al. 1999). Therefore, we were not able to analyze copper transporter expression in relation to copper concentration within enterocytes. Whether diminished CTR1 expression and augmented ATP7A expression in the intestine of WND patients is the local response to high copper levels or is stimulated by the hormone(s) or the other systemic signals remains to be evaluated in further studies.

Irrespective of the stimulus or mechanism causing variable copper transporter expression, we expected differences between treated and untreated patients. We observed reduced expression of mRNA for *CTR1* and increased transcription activity of *ATP7A* in treatment naive patients in comparison to patients on de-coppering treatment; however, there was no such difference in protein expression. The WND treatment group patients were not homogenous. The group comprised subjects treated with penicyllamine or zinc salts; moreover, the patients differ in terms of the duration of treatment, which ranged from one week to years. The group was too small to enable a separate analysis of the influence of treatment duration and drug type on copper transporter expression. For the duodenal biopsy we used standard forceps for 2.8 mm biopsy channels, which can retrieve a full-thickness biopsy of the mucosa and occasionally a small amount of submucosa. The mucosal layer of the gut consists of epithelial cells, mostly enterocytes overlying lamina propria or connective tissue core resting on a layer of smooth muscle. The submucosa is composed of connective tissue and vascular structures. It was not possible to obtain the same thickness of biopsy from each study participant; thus, the samples varied in cellular content. The heterogeneity of the studied groups and the variable duodenal sample size and lower sensitivity of the Western blot in comparison to PCR are potential explanations for the failure of Western blot in the detection of quantitative differences in CTR1 and ATP7A expression between the two populations of WND patients.

We did not observe any significant differences in the expression of DMT1 between WND and the controls. Notwithstanding the fact that DMT1 is capable of transporting copper in vitro (Mackenzie and Hediger 2004), our observation is in accordance with the commonly accepted notion of a limited DMT1 role in copper transport in humans (Garrick et al. 2006).

Neither did we detect differences in ATP7B expression or localization between WND patients and controls. This observation was slightly surprising as many *ATP7B* mutations cause instability and mislocalization of the protein. In 69 out of the 109 WND patients, we detected H1069Q mutation in at least one allele of *ATP7B*. It has been reported that *ATP7B* mutations do not change mRNA expression significantly (van den Berghe et al. 2009). The published data on H1069Q-ATP7B protein stability are not conclusive; however, recent in vitro studies have proved that H1069Q mutation does not alter the structure nor folding of ATP7B (Payne et al. 1998, Tsivkovskii et al. 2003, Dmitriev et al. 2006, Dmitriev et al. 2011). There are also controversies regarding the half-life of wild type ATP7B which, depending of cell type, varies from 8 to 22 h (Payneet al. 1998, van den Berghe et al. 2009). Nevertheless, even if the half-life of mutant and wild type protein differs in vivo, it would be difficult to detect the difference in ATP7B expression as the average life span of an enterocyte is short, approximately 4–5 days. Moreover, the antibody we used was polyclonal and raised against the N-terminus of the protein; therefore, it could potentially bind to already defragmented ATP7B. The mutant H1069Q-ATP7B is mislocalized in the endoplasmatic reticulum, while the wild type protein resides in the trans Golgi network (Huster et al. 2003). In the intestine specimens, we observed cytoplasmatic, perinuclear expression of ATP7B both in patients and controls. The immunohistochemical technique we used did not allow us to differentiate between endoplasmatic and trans Golgi localization.

As we did not observe any significant differences in ATP7B expression between WND patients and controls, we can only speculate on the role of ATP7B in copper homeostasis within enterocytes. In the murine intestine, ATP7B was localized to small vesicles within the cytoplasm (Weiss et al. 2008). ATP7B has been postulated to support copper efflux at the apical membrane or store excessive copper in the vesicles. If the main role of ATP7B in the enterocyte is sequestration of excess copper and copper excretion on the luminal side (as in hepatocytes), the increased ATP7A production in WND could be a backup mechanism in response to the malfunction of ATP7B.

Based on the study results, we were not able to evaluate if different expressions of the copper transporter in the duodenum of WND patients influence net copper absorption from the intestine. We did not find any correlation between duodenal expression of the studied transporters and serum markers of copper metabolism. However, neither serum ceruloplasmin nor total serum copper concentration reflects copper absorption from the intestine.

A merit of our study is the ethnic homogeneity of our population as well the homogenous methodology of clinical data collection. However, our study has some limitations. WND patients and control groups differed in terms of the age of participants and sex distribution. In regression analysis neither age or sex influenced mRNA for either CTR1 or ATP7A expression; thus, this limitation did not significantly affect our observations.

In conclusion, we demonstrated augmented ATP7A expression and diminished CTR1 expression in duodenal samples of WND patients, which can be interpreted as an enterocyte compensatory mechanism to systemic copper overload. The increased *ATP7A* transcription may enhance copper excretion and/or sequestration, while diminished CTR1 production may protect against the copper influx. It remains to be explored in further studies as to whether Sp1 or other mediators regulate copper transporter expression in human enterocytes. Whether the modification of copper transporter expression in the intestine may represent a new therapeutic target in WND remains to be elucidated. However, it is a promising strategy to overcome drug resistance in neoplastic diseases (Gupta and Lutsenko 2009). It has been documented that ATP7A, ATP7B and CTR1 transport platinum–based antitumor agents, and down-regulation of CTR1 and upregulation of copper ATPases is linked with tumor resistance to chemotherapy (Holzer et al. 2004, Katano et al. 2004, Samimi et al. 2004). Recent findings on copper-controlled CTR1 expression have initiated a phase I clinical trial assessing the usefulness of therapy with a copper chelator (trientine) as an enhancer in advanced ovarian cancer chemotherapy based on carboplatin (Fu et al. 2012).
